# Association of clinical and demographic factors with fungal culture in pulmonary tuberculosis patients

**DOI:** 10.6026/973206300220452

**Published:** 2026-01-31

**Authors:** Priyanka Prasad, Suneel Kumar Ahirwar, Deepak Bansal, Anju Mahor, Manish Purohit

**Affiliations:** 1 Department of Microbiology, MGM, Medical College Indore, Madhya Pradesh, India

**Keywords:** Pulmonary tuberculosis, fungal co-infection, risk factors, aspergillus, candida, fungal culture

## Abstract

Pulmonary fungal infections are a significant co-infection in patients with pulmonary tuberculosis (PTB). Therefore, it is of interest to examine the relationship between clinical and demographic factors and fungal culture positivity in suspected or confirmed PTB cases. A cross-sectional study of 180 individuals was conducted at MGM Medical College, Indore, using sputum samples for fungal culture and KOH mount. Fungal positivity was found in 20% of patients, with a significant association with proven PTB (p = 0.003). Thus, the significant association between pulmonary tuberculosis and fungal co-infections, emphasizing the need for routine fungal screening to improve diagnosis and clinical outcomes is reported.

## Background:

Pulmonary tuberculosis (PTB) is a major global public health concern, particularly in developing countries like India, which accounts for roughly a quarter of the global TB burden [1]. Despite breakthroughs in detection and treatment, tuberculosis (TB) still causes significant morbidity due to structural lung damage and immunological dysregulation [2]. These pathological alterations make patients susceptible to secondary infections, particularly lung fungal infections. Fungal infections of the lungs are increasingly recognized as major opportunistic infections in PTB patients, frequently affecting disease progression and treatment results [3]. The common clinical symptoms of tuberculosis and pulmonary fungal infections, such as chronic cough, hemoptysis, fever, weight loss and radiological cavitary lesions, usually result in misdiagnosis or delayed diagnosis [4]. Aspergillus species, notably Aspergillus fumigatus, are the most common fungal infections linked to post-tubercular lung illness because of their capacity to inhabit pre-existing cavities [5]. Candida species, while considered commensals, can act as opportunistic infections in immunocompromised tuberculosis patients [6]. Several studies in India have found fungal co-infection rates ranging from 14% to 30% in PTB patients [7]. Male gender, smoking, rural residency, diabetes mellitus and chronic obstructive pulmonary disease (COPD) have all been recognized as significant risk factors for fungal colonization and infection in tuberculosis patients [8]. However, data on the combined effect of these factors on fungal culture positive in PTB patients are scarce. Hence, early detection of fungal co-infection is critical, since untreated fungal disease can result in chronic symptoms, treatment failure and increased mortality. Therefore, it is of interest to investigate the relationship between clinical and demographic characteristics and fungal culture positivity in suspected and confirmed pulmonary tuberculosis cases.

## Materials and Methods:

A cross-sectional observational study was conducted in the Department of Microbiology in collaboration with the Department of Respiratory Medicine at MGM Medical College, Indore, India. A total of 180 patients with suspected or confirmed pulmonary tuberculosis attending outpatient and inpatient services were included.

## Inclusion criteria:

[1] Patients clinically suspected of pulmonary tuberculosis

[2] Microbiologically confirmed PTB cases

[3] Patients willing to provide informed consent

## Exclusion criteria:

[1] Patients already on antifungal therapy

[2] Inadequate or contaminated sputum samples

## Sample collection:

Early morning sputum samples were collected in sterile, wide-mouthed containers following proper oral hygiene measures. Samples were transported promptly to the laboratory for processing.

## Laboratory processing:

Direct Microscopy: Sputum samples were examined using 10-20% potassium hydroxide (KOH) mount for the presence of fungal elements.

## Fungal culture:

Samples were inoculated on Sabouraud dextrose agar with and without antibiotics and incubated at 25°C and 37°C for up to four weeks. Growth was monitored periodically.

## Identification of fungi:

Fungal isolates were identified based on colony morphology and microscopic characteristics using lactophenol cotton blue mount, slide culture, germ tube test, CHROM agar and Dalmau plate method where applicable.

## Data collection:

Demographic details (age, sex, residence and occupation), clinical features, smoking history, TB status and associated comorbidities were recorded using a predesigned proforma.

## Statistical analysis:

Data were analyzed using standard statistical software. Associations between fungal culture positivity and various factors were assessed using chi-square test, with p < 0.05 considered statistically significant.

## Results:

**Figure 1:**
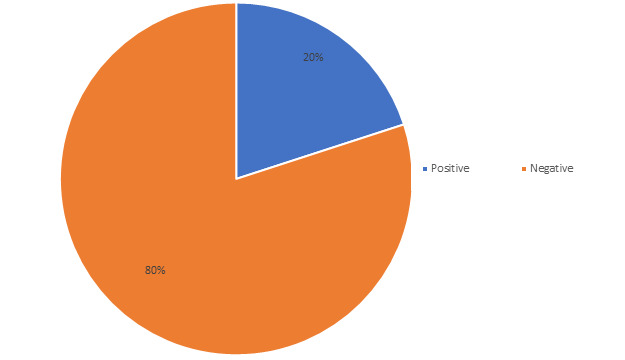
Fungal culture positivity in study population

A total of 180 patients with suspected and confirmed pulmonary tuberculosis were included in the study. Fungal culture positivity and its association with clinical and demographic factors were analyzed. Fungal culture positivity rates were 20% among pulmonary tuberculosis patients (Figure 1). Fungal culture positivity showed no statistically significant association with gender, age group, residential area, or occupation. Slightly higher positivity was observed among females, patients aged 41-60 years, rural residents and skilled workers; however, these differences were not significant (Table 1). A statistically significant association was observed between TB status and fungal culture positivity (p = 0.0034), with TB-positive patients showing higher fungal isolation rates. No significant association was found between fungal culture positivity and smoking history or presence of comorbidities (Table 2). Table 3 illustrates the association between various demographic and clinical variables and fungal culture positivity among patients with suspected or confirmed pulmonary tuberculosis.

## Discussion:

Pulmonary tuberculosis is a significant risk factor for subsequent pulmonary fungal infections due to prolonged lung damage, cavitary lesions and compromised host immune responses. In the current investigation, fungal culture positive was found in 20% of patients with suspected and confirmed pulmonary tuberculosis, highlighting the clinical importance of fungal co-infection in TB-endemic areas. The prevalence of fungal culture positivity in our study is consistent with data from numerous Indian and international investigations, where fungal co-infection rates among pulmonary tuberculosis patients ranged from 15% to 30% [9]. Such variation may be due to changes in environmental exposure, meteorological circumstances, patient demographics and laboratory diagnostic methods. In the current investigation, a substantial relationship was found between proven pulmonary tuberculosis and positive fungal cultures. Patients with microbiologically confirmed tuberculosis had a greater risk of fungal isolation. Xue *et al.* reported similar findings, emphasizing that structural lung damage caused by tuberculosis creates an ideal environment for fungal colonization and infection, notably by Aspergillus species [10]. Several investigations have found a substantial relationship between chronic inflammation and residual cavities after TB treatment and pulmonary mycoses [11]. Although fungal culture positive was more common in male patients, the relationship was not statistically significant. Male preponderance has been documented in various researches and is frequently linked to increased environmental exposure, occupational risk and higher smoking prevalence rather than innate biological sensitivity [12]. The lack of statistical significance in the current study implies that gender alone may not be a reliable predictor of fungal co-infection. Smoking is recognized as a factor that impairs lung defense mechanisms and mucociliary clearance. However, the present investigation found no statistically significant link between smoking and fungus culture positive [13]. Differences in smoking intensity and duration may explain why studies provide conflicting results. In the current investigation, fungal culture-positive individuals were more likely to have comorbid diseases such as diabetes mellitus and chronic obstructive pulmonary disease; however this was not statistically significant. Diabetes-related immunological dysfunction has been extensively studied as a risk factor for fungal infections [14]. Several investigations have found a higher prevalence of fungal infections among diabetic tuberculosis patients, albeit statistical significance has not been consistently established [15]. Patients in rural locations had modestly higher fungal culture positive, which was most likely owing to increased exposure to environmental fungus found in soil, agricultural settings and organic debris [16]. Occupational study also revealed increased fungal isolation among skilled workers and unemployed individuals, though not statistically significant, implying that occupational exposure alone may not predict fungal infection. A multivariate logistic regression analysis found that proven tuberculosis status was the only independent predictor of fungal culture positivity. This observation underlines the essential role that pulmonary tuberculosis plays in predisposing individuals to secondary fungal infections, as previously documented [17]. Overall, the findings highlight the necessity of raising clinical suspicion and performing routine fungal testing in patients with pulmonary tuberculosis, particularly those with proven illness and persistent respiratory symptoms.

## Conclusion:

Patients with pulmonary tuberculosis frequently develop fungal co-infection. Confirmed tuberculosis was revealed to be the only independent predictor of positive fungal cultures. Males, middle-aged patients, rural dwellers, smokers and those with comorbidities had greater fungal isolation rates, although these differences did not reach statistical significance. Routine fungal screening should be considered in individuals with confirmed pulmonary tuberculosis to allow for early detection and better patient outcomes.

## Figures and Tables

**Table 1:** Association of demographic factors with fungal culture positivity

**Variables**		**Number of Patient**	**Culture positive**	**P-Value**
Age Group (Years)	≤20	15 (8.3%)	3 (20%)	0.958
	21-40	75 (41.7%)	13 (17.3%)	
	41-60	64 (35.5%)	15 (23.4%)	
	61-80	25 (13.9%)	5 (20%)	
	>80	1 (0.6%)	0 (0%)	
Gender	Male	108 (60%)	19 (17.6%)	0.424
	Female	72(40%)	17 (23.6%)	
Residential Area	Urban	79 (43.9%)	16 (20.3%)	0.951
	Rural	101 (56.1%)	20 (19.8%)	
Occupation	Professional	10 (5.6%)	0 (0%)	0.295
	Semi-Professional	18 (10%)	0 (0%)	
	Semi-skilled worker	35 (19.4%)	2 (5.7%)	
	Skilled worker	41 (22.8%)	6 (14.6%)	
	Unskilled worker	20 (11.1%)	1 (5%)	
	Unemployed	56 (31.1%)	7 (12.5%)	

**Table 2:** Association of clinical factors with fungal culture positivity

**Clinical Factor**	**Culture Positive N (%)**	**Culture Negative N (%)**	**P-Value**
TB Positive	16 (23.2%)	53 (76.8%)	0.003
TB Negative	20 (18.0%)	91 (82.0%)	
Smoking (Yes)	5 (15.2%)	28 (84.8%)	0.596
Smoking (No)	31 (21.1%)	116 (78.9%)	
Comorbidity (Yes)	7 (15.9%)	37 (84.1%)	0.573
Comorbidity (No)	29 (21.3%)	107 (78.7%)	

**Table 3:** Association of clinical and demographic factors with fungal culture positivity among suspected and confirmed pulmonary tuberculosis patients

**Variable**	**Culture Positive n (%)**	**Crude OR (95% CI)**	**Adjusted OR (95% CI)**	**P-Value**
TB status (Positive)	16 (69)	1.37 (1.04-2.46)	1.38 (1.06-2.51)	0.003
Comorbidity (Yes)	7 (44)	0.7 (0.28-1.73)	0.73 (0.29-1.83)	0.573
Smoking (Yes)	5 (33)	0.67 (0.24-1.87)	0.82 (0.27-2.48)	0.596
Gender (Male)	19 (108)	0.69 (0.33-1.44)	0.73 (0.33-1.58)	0.424
Age group 41-60 yrs	15 (64)	1.38 (0.66-2.92)	1.54 (0.71-3.36)	0.508
TB-positive with Comorbidity	3 (17)	0.84 (0.23-3.11)	0.81 (0.21-3.06)	1
